# Genetic and Epigenetic Aberrations of *SOX7* in Newly Diagnosed and Relapsed Multiple Myeloma as Well as Related Neoplasms

**DOI:** 10.3390/cimb47040244

**Published:** 2025-04-01

**Authors:** Can Küçük, Burcu Akman, Xiaozhou Hu, Tevfik Hatipoğlu, Ahmet Şeyhanlı, Arda Ceylan, Bircan Yılmaz, Osman Can Öztürk, Taner Kemal Erdağ, Güner Hayri Özsan

**Affiliations:** 1 Department of Medical Biology, Faculty of Medicine, Dokuz Eylül University, 35340 İzmir, Türkiye; 2 İzmir International Biomedicine and Genome Institute, Dokuz Eylül University, 35340 İzmir, Türkiye; 3 İzmir Biomedicine and Genome Center, 35340 İzmir, Türkiye; 4 Department of Medical Biochemistry, Faculty of Medicine, Dokuz Eylül University, 35340 İzmir, Türkiye; 5 Department of Hematology, Faculty of Medicine, Dokuz Eylül University, 35340 İzmir, Türkiye; 6 Department of Hematology, Faculty of Medicine, Gazi University, 06560 Ankara, Türkiye; 7 Department of Otorhinolaryngology, Faculty of Medicine, Dokuz Eylül University, 35340 İzmir, Türkiye

**Keywords:** *SOX7*, deletion, promoter methylation, biomarker, multiple myeloma, relapse, plasma cell neoplasms

## Abstract

Multiple myeloma (MM) is one of the most frequent hematological malignancies. Most MM cases relapse, which is associated with poor prognosis. MM-related tumor suppressor genes are not totally known yet. *SOX7* is one of the tumor suppressor candidates located in 8p23.1, a recurrently deleted region in MM. Here, we evaluated the genetic and epigenetic aberrancies of *SOX7* in diagnostic or relapsed MM as well as smoldering MM (SMM) and plasma cell leukemia (PCL). Publicly available datasets were reanalyzed to evaluate *SOX7* copy number, promoter methylation, transcript levels in MM or related neoplasms and to evaluate mutation rates in MM cases. qPCR and qRT-PCR with an in-house MM cohort were performed to cross-validate *SOX7* copy number and transcript level estimates. *SOX7* deletions were frequent in newly diagnosed and relapsed MM cases. *SOX7* promoter hypermethylation was observed in MM cell lines, MM cases, and PCL cases. Importantly, *SOX7* was transcriptionally silent in MM cell lines and underexpressed in MM and high-risk SMM cases. Integrative analyses of patient-matched diagnostic and relapsed MM tumor tissues revealed moderate positive correlations between *SOX7* copy numbers. *SOX7* deletion and promoter methylation levels had a tendency to be mutually exclusive. *SOX7* promoter methylation levels were significantly higher in relapsed cases compared to the diagnostic ones. *SOX7* mutations were rare in MM cases. *SOX7* underexpression may be due to genetic and/or epigenetic alterations in newly diagnosed and relapsed MM. These genetic and epigenetic aberrations can serve as diagnostic or prognostic biomarkers for MM and allied neoplasms. Future research will reveal whether *SOX7* inactivation has a role in development of these plasma cell neoplasms.

## 1. Introduction

Multiple myeloma is an incurable type of lymphoid cancer that develops as a result of the clonal proliferation of antibody-producing plasma cells in the bone marrow [1]. Clonal plasma cells in bone marrow of MM patients express CD138 and CD38 on their surface and lack expression of CD19 [2]. MM incidence and mortality rate have a globally increasing trend [3]. As a frequently observed hematological malignancy, it is responsible for ~2% of the cancer-related deaths [4]. It originates from smoldering multiple myeloma (SMM), which is a benign, asymptomatic neoplasm [5]. MM may transform into plasma cell leukemia (PCL), a more aggressive type of plasma cell malignancy [6].

The drugs effective for the treatment of MM mainly include bortezomib [7], as well as immunomodulatory drugs such as lenalidomide [8] or thalidomide [9]. Ten-year survival of MM patients doubled compared to the 1970s with the inclusion of these drugs in the early 2000s [10]. However, a relapse is observed in most patients after these treatments [11], which is associated with poor survival rates [12,13]. Consequently, the identification of molecular aberrations leading to therapy resistance is highly important for the development of effective therapeutics against relapsed MM patients.

Genetic and epigenetic alterations were proposed as diagnostic biomarkers for several different cancer types [14,15]. Genomic deletions are among genetic alterations that were shown to have diagnostic biomarker potential in certain cancer types such as non-small-cell lung cancer [16]. Among epigenetic biomarkers, several studies indicated diagnostic potential for DNA methylations, including but not limited to colorectal [17] or lung cancer [18]. Genetic and/or epigenetic diagnostic biomarkers can potentially be used to improve molecular diagnosis of MM and related plasma cell neoplasms.

Tumor suppressor genes located in recurrently deleted regions contributing to MM development and relapse have not been fully identified yet. In a comprehensive genome-wide study, copy number alterations of 153 MM patient samples were determined with Affymetrix SNP 6.0 arrays [19]. Interestingly, homozygous deletions of the 8p23.1 genomic locus that includes tumor suppressor candidates were identified in this study [19]. This bi-allelically deleted locus contains three tumor suppressor gene candidates (*MSRA*, *PINX1*, *SOX7*) [20,21,22].

Among these three tumor suppressor gene candidates in del8p23.1, a higher number of research articles supported the role of *SOX7* as a tumor suppressor in a variety of cancer types compared to other tumor suppressor candidates. As a transcription factor, *SOX7* was reported to transcriptionally regulate several genes related to its tumor suppressor functions in breast cancer cells [23]. In a study on colorectal cancer (CRC), *SOX7* was shown in CRC cell lines and tumor tissues to be transcriptionally silenced through abnormal DNA methylation. In the same article, ectopic expression of *SOX7* in CRC cells promoted apoptosis and inhibited proliferation [22]. *SOX7* expression was reported to be silenced in tumor tissues and cell lines in non-small-cell lung cancer in another study, which also showed inhibition of cell growth and promotion of apoptosis after re-expression of *SOX7* [24]. Importantly, reduced *SOX7* expression in lung cancer cells was shown to be associated with shortened survival and lymph node metastasis [25]. The tumor-suppressive effects of *SOX7* may occur through the regulation of activities in different signaling pathways, primarily Wnt/β-catenin. In gastric cancer tumors, *SOX7* silencing was demonstrated to lead to dysregulated activation of the Wnt/β-catenin signaling pathway [26]. Similarly, *SOX7* inactivation causing dysregulated activation of the Wnt/β-catenin signaling pathway was also reported in ovarian cancer [27]. The reason of *SOX7*-induced apoptosis in various cancer types may be owing to its activation of the MAPK/ERK-BIM apoptotic signaling pathway [28].

To the best of our knowledge, there has been no study performed until now investigating the presence of genetic or epigenetic aberrations specifically of *SOX7* in newly diagnosed and relapsed MM besides related neoplasms (i.e., SMM and PCL). Based on the observations in other cancer types supporting the role of *SOX7* as a tumor suppressor as well as a recent study showing *SOX7* deletion in an MM case with double relapse [29], the genetic and/or epigenetic aberrations of *SOX7* may be responsible for transcriptional downregulation of *SOX7* in MM and allied neoplasms. In this study, we investigated the copy number status and promoter methylation levels of *SOX7* in diagnostic or relapsed MM in addition to related neoplasms through different approaches with generally consistent results. We also provided evidence that deletion may decrease mRNA expression of *PINX1* and *MSRA,* which are the other two tumor suppressor candidates located on del8p23.1.

## 2. Materials and Methods

### 2.1. Patient Information

Fifteen newly diagnosed and thirteen relapsed MM cases were included as the in-house patient cohort in this study. The summary of clinicopathologic and demographic characteristics of these MM cases is available in Table S1 for these newly diagnosed and relapsed MM cases.

### 2.2. Chemicals and Reagents

Ficoll-Paque Plus (cat. no: GE17-1440-02) was purchased from Cytiva (Uppsala, Sweden), and 10x PBS (cat. no: 70011-036) was obtained from Thermo Fisher Scientific (Waltham, MA, USA). A total of 500 ml of 10x ACK lysing buffer (pH 7.2) was prepared by dissolving 43 g 1.5 M ammonium chloride (NH_4_Cl) (Kimetsan, Ankara, Türkiye), 5 g 100 mM potassium bicarbonate (KHCO_3_) (Merck, Darmstadt, Germany), and 10 mM Triplex111 (EDTA-2Na) (Kimetsan, Ankara, Türkiye). DAPI (cat. no: 422801) was purchased from BioLegend (San Diego, CA, USA). β-Mercaptoethanol (cat. no: M6250-100 ML, purity: ≥99.0%) was purchased from Sigma-Aldrich (St. Louis, MO, USA) to be used in DNA and RNA isolations with the AllPrep DNA/RNA isolation kit (Qiagen, Hilden, Germany). dNTP mix (cat. no: N0447S), 100 bp DNA ladder (cat. no: N3231S) as well as Taq DNA polymerase and buffer (cat. no: M0273S) were purchased from New England Biolabs (Ipswich, MA, USA). Ethidium bromide (cat no: 4410-10ML) and concentrated TAE buffer solution (cat. no: TAE222.1) were purchased from Millipore (Waltham, MA, USA) and BioShop Canada (Burlington, ON, Canada), respectively.

### 2.3. FACS Sorting of Bone Marrow Tumor Cells

Mononuclear cells were separated from the MM patient bone marrow samples either through the Ficoll-Paque Plus method or through red blood cell lysis by addition of 1x ammonium–chloride–potassium (ACK) lysing buffer solution. Before deciding the amount of bone marrow and antibody amount to be used, test staining was performed according to the manufacturer’s recommendations by adding 5 µL of each antibody to 100 µL of bone marrow samples. The antibodies used in this study were anti-CD138-FITC, anti-CD38-PE, and anti-CD19-APC antibodies (BD Biosciences, San Jose, CA, USA). After that, cells were resuspended in 1x phosphate-buffered saline (PBS) followed by quantification of the percentages of tumor cells with the BD FACSCanto II instrument. In this FACS test, cells were gated based on the FSC and SSC values. Single cells (singlets) were selected followed by gating of the cells with the CD19^−^ phenotype. Another gating was performed in the CD19^−^ cell population for CD138^+^ and CD38^+^ phenotype cells so that the percentages of CD138^+^/CD38^+^/CD19^−^ cells [30] were determined among all cells.

Bone marrow and antibody amounts to be used in FACS sorting were determined based on the calculated tumor cell percentages and total cell numbers. When the tumor cell percentages were sufficient, the experimental procedures were repeated the same way as above by sorting cells with the BD FACSAria^™^ III Cell Sorter. FACS sorting was not performed for the corresponding MM case when tumor cell numbers were low.

### 2.4. Isolation of DNA and RNA from MM Bone Marrow Tumor Cells

DNA and RNA were simultaneously isolated from MM bone marrow tumor cells with CD138^+^/CD38^+^/CD19^−^ phenotype using AllPrep DNA/RNA Mini Kit (Qiagen Inc., Hilden, Germany) per manufacturer’s instructions. AllPrep DNA/RNA Micro Kit (Qiagen Inc., Hilden, Germany) was used when the FACS-separated cell numbers were low. To maximize the DNA and RNA yields, elution steps were repeated in different collection tubes.

### 2.5. Quality Control of the MM Bone Marrow Tumor Cell DNA and RNA

The initial quality check (QC) of the MM bone marrow tumor DNA and RNA samples was performed with NanoDrop 2000 spectrophotometer (Thermo Fisher Scientific, Waltham, MA, USA) to evaluate the 260/280 ratios to ensure lack of protein contamination, as well as the 260/230 ratios to ensure a lack of contamination from solvents, salts, or carbohydrates used during extraction. As MM BM DNA and RNA sample amounts were generally low, PCR-based methods were used for a secondary QC. The quality control for genomic DNA samples was performed with PCR by using the REL ex11-5 primer pair that was available from a previous study [31]. The specificity and size of the amplicons were evaluated by imaging the PCR products that were run in the TAE gel. As expected, 271 bp bands were obtained with the REL ex11-5 primer pair PCR amplifications. The QC of total RNA samples was performed with reverse transcriptase PCR (RT-PCR) experiments by using QuantiTect Reverse Transcription Kit (Qiagen Inc., Hilden, Germany). During reverse transcription reactions, ~30 ng RNAs were converted to cDNA biomolecules. Obtained cDNAs were then 1:10 diluted with RNase-free H_2_O for the subsequent PCR experiments. After that, PCRs were performed by using an RPL13A qRT-PCR primer pair designed in an exon–exon junction with an expected amplicon size of 149 bp.

### 2.6. Tonsil Samples from Control Cases

As control group samples, plasma cells (PCs) obtained through FACS from reactive tonsil tissues of tonsillitis (*n* = 3) or obstructive sleep apnea (*n* = 5) patients were used in this study. Based on previous reports describing CD38 bright (CD38^++^) cells in reactive tonsil tissues as PCs [32,33], CD38^++^ tonsil cells were sorted with flow cytometry as PCs. Fresh tonsil tissue samples used as control samples were obtained through routine tonsillectomy operations at the Department of Otorhinolaryngology in DEU. Half of the tonsil tissue pairs were delivered to the Department of Medical Pathology at DEU for sample registration. The remaining half of the tonsil tissues were placed into 1x PBS solution with/without EDTA for subsequent procedural steps. The tonsil tissues were processed as follows until FACS cell separations: Tonsil tissues were fractionated and homogenized using forceps followed by suspension of cells in 1x PBS as 50 mL volume. For the elimination of cell clumps and debris, tonsil cell suspensions were passed through 100 µm-pored cell filters (Corning Inc., Corning, NY, USA, cat no: 352360). After the addition of 15 mL Ficoll-Paque solution into 20 mL tonsil cell suspension, cells were centrifuged at 1000× *g* speed for 20 min at room temperature. After the centrifuge, the mononuclear cells present in the interphase were collected and counted by trypan blue exclusion assay.

Tonsil samples from 5 obstructive sleep apnea cases were prepared for FACS cell separations as follows: 50 mL cell suspensions were prepared inside 1x PBS + 5 mM EDTA solution after fractionation and homogenization of tonsil tissues with forceps. Tonsil cell suspensions were filtered through Falcon^®^ 100 µm Cell Strainer (Corning Inc., cat no: 352360) to eliminate cell clumps and debris. Then, cell suspensions were incubated for 15 min inside 1x ACK lysis buffer solution added in a 1:4 ratio. Cells were centrifuged twice, the latter of which included the addition of 1x PBS + 5 mM EDTA solution for washing. Tonsil cells were then suspended in 20 mL of 1x PBS + 5 mM EDTA. After that, the number of tonsil cells was determined with the hemocytometer using a trypan blue exclusion assay.

### 2.7. Tonsil PC Separations Through FACS

After the determination of cell numbers, tonsil cells were stained with CD38-PE antibody and prepared for flow cytometry. In the first stage, 3 µL CD38-PE (Biolegend, San Diego, CA, USA) antibody was added into 100 µL volume cell suspension and incubated for 20 min in the dark. The cells were then washed by the addition of 2 mL 1x PBS followed by centrifugation at 400× *g* for 10 min at room temperature. After the removal of the supernatant, the cell pellet was resuspended in 500 µL of 1x PBS. Five microliter DAPI (50 µg/mL) was added to label dead cells. CD38-stained cells were analyzed with flow cytometry, and CD38^++^ (CD38 bright) cell ratios were determined. With this FACS test, PC ratios were determined among tonsil cells analyzed before the cell sorting process. For the cell sorting processes, 3.5 mL cell suspension was incubated with 100 µL CD38-PE and then washed with 1x PBS. After that, CD38^++^ phenotype cells were sorted with flow cytometry.

### 2.8. SOX7 Copy Number Analyses of Publicly Available SNP Array Data of Diagnostic MM Cases

Publicly available Affymetrix GeneChip Human Mapping 250K Nsp array data available in NCBI GEO database (accession no: GSE21349) were reanalyzed for 40 diagnostic MM tumor samples as well as patient-matched white blood cell (WBC) samples to evaluate the copy number status of *SOX7*. In this dataset, diagnostic MM tumor samples were pre-treatment CD138^+^ PC samples that were positively selected using MACS beads, and then stored in RLT buffer. DNA isolations were performed with the Qiagen AllPrep kit per the manufacturer’s instructions. Peripheral white blood cell pellets of the same MM patients were used as normal controls with respect to *SOX7* copy number status. Genomic DNA of WBC samples was extracted from peripheral white blood cell pellets using the Qiagen FlexiGene kit, as per the manufacturer’s instructions. A total of 250 ng of DNA per each sample was digested with NspI/StyI and processed according to the Affymetrix 500K protocol. DNA samples were end-labeled with biotin using TdT. In all, 90 µg of fragmented labeled DNA samples were hybridized to the array for 16 h at 49 °C according to Affymetrix protocols. Genechips were scanned on an GeneChip Scanner 3000 7G/4 color (Early Access) (Affymetrix, Santa Clara, CA, USA) and processed using GCOS 1.4.0. SNP genotype calls were generated using Affymetrix Powertools v1.8.0 (apt-probeset-genotype). Calls were generated using the default parameters and the brlmm calling algorithm.

During *SOX7* copy number analyses, values of each of the two different probesets for *SOX7* (SNP_A-1859173 and SNP_A-1886065) were divided into the probeset values of *RPL37A* (SNP_A-1913649) followed by normalizations of *SOX7* values in MM tumor samples to patient-matched WBC samples. MM tumors with copy number values ≤ 75% of the corresponding WBC samples for each *SOX7* probeset were considered to have *SOX7* deletion.

### 2.9. SOX7 Deletion Analyses in Diagnostic and Relapsed MM Cases with qPCR

The presence of *SOX7* deletions was evaluated with qPCR in MM patient tumor samples. For normalization of *SOX7* copy numbers, *EMC7* and *RPL37A* genes that reside, respectively, in chromosomes 15 and 2 were chosen. Trisomy or gains of chromosome 15 were observed in some MM samples based on previous publications [34], whereas no chromosomal abnormality was detected for chromosome 2 that may generate false positive results. Therefore, MM cases with *SOX7* copy numbers ≤ 0.75-fold of those of the average of control samples based on normalizations for both *EMC7* and *RPL37A* were considered to have *SOX7* deletion. Primer pairs for qPCR were designed with the PrimerQuest software (IDT DNA Technologies, Coralville, IA, USA) and then ordered from Sentebiolab Biotech (Ankara, Türkiye). Different primer pairs of *SOX7* and *EMC7* were optimized with gradient PCR for qPCR analyses. qPCR experiments were repeated twice. Copy numbers were evaluated with the ddCt method. Primers used for gene copy number analyses are shown in Table S2A.

### 2.10. Promoter Methylation Analyses of SOX7 Using Publicly Available Methylation Array Data of MM and PCL

*SOX7* promoter methylation analyses of MM and PCL cases were performed by reanalyzing methylation profiling data publicly available and previously published [35] in the context of GSE21304 in the NCBI GEO database. This series includes methylation profiling data based on the HumanMethylation27_270596_v.1.2 BeadChip (Illumina San Diego, CA, USA) for 161 MM cases, 7 PCL cases, and 9 MM cell lines. Briefly, this dataset was generated as follows: PCs from non-myeloma patients (normal plasma cell controls, *n* = 3) and MM patients (*n* = 161) were selected to a purity of >90% using CD138 microbeads and MACS (Miltenyi Biotech, Bisley, UK). Samples from PCL patients (*n* = 7) were not CD138 selected; however, they had >90% PC infiltration as determined by microscopy. Some normal PC control samples were pooled to obtain a sufficient quantity of DNA. B cells (*n* = 6) from normal individuals were selected from peripheral blood using CD19. Genomic DNA biomolecules were extracted using the AllPrep protocol (Qiagen, Hilden, Germany). For labeling, hybridization, and scanning of bisulfite-modified genomic DNA, the manufacturer Illumina’s instructions were followed. For data processing, files were analyzed in GenomeStudio (Illumina, San Diego, CA, USA) as per manufacturer’s (Illumina) instructions using the methylation module v1.5.5 and were described as beta values.

*SOX7* promoter methylation levels were analyzed using an approach similar to a previous publication [28] by determining the cg08056146 probe intensity levels through the ‘Profile graph’ option of the GEO2R bioinformatic tool. Methylation profiling data of peripheral blood B cell samples (*n* = 6) and PC samples (*n* = 3) from normal individuals available in this GEO series were used as controls.

### 2.11. Integrated Analyses of Copy Number, Promoter Methylation, and Transcript Expression of the Candidate Tumor Suppressors Located in del8p23.1 in Diagnostic and Relapsed MM Cases

Publicly available NCBI GEO SuperSeries (GSE77540) associated with a previously reported study [36] was used for integrated analyses of *SOX7*, *PINX1*, and *MSRA* copy number, promoter methylation, and transcript expression in the same MM tumor samples belonging to patient-matched diagnostic and relapsed MM cases.

For DNA copy number analyses, CD138^+^ PCs from the bone marrow of MM patients during diagnosis and relapse stages were collected. Total RNA from these cells was extracted using Buffer RLT plus (Qiagen, Hilden, Germany), followed by DNA and RNA purification with the AllPrep DNA/RNA Mini Kit (Qiagen, Hilden, Germany). Labeling and hybridizations for the CytoScan 750K Array (Affymetrix, Santa Clara, CA, USA) were performed according to Affymetrix protocol. The hybridization protocol included restriction digestion of DNA, PCR amplification, fragmentation, labeling, and hybridization of each array according to the manufacturer’s instructions. The scanning protocol consisted of an array washing step using Affymetrix fluidics stations and scanning using the GeneChip Scanner 3000. For data processing, the ChAS console from Affymetrix was used to assess genome-wide copy number frequencies. The normalized data matrix contained weighted log2 ratios. The *cychp files were generated as follows: affymetrix-algorithm-name = CYTO2, affymetrix-algorithm-version = 1.16.0 program-name = apt-copynumber-cyto.exe program-version = 1.16.0. Normalized probeset values for *SOX7*, *PINX1*, and *MSRA* genes generated with the Affymetrix CytoScan 750K Array were used to evaluate the copy number of these genes in 19 patient-matched diagnostic and relapsed MM tumor samples. MM cases with values ≤ 0.75 were considered to have a deletion of the gene of interest. The probeset IDs for *SOX7*, *PINX1*, and *MSRA* genes are, respectively, as follows: C-6DUBL, C-4YZRZ, and C-4CMDY.

For DNA methylation analyses, genomic DNA from CD138^+^ PCs was extracted from frozen bone marrow samples with QIAmp DNA Mini Kit (Qiagen, Valencia, CA, USA) following the manufacturer’s instructions. Samples were denatured and labeled in parallel with Cy3 for the test group and Cy5 for the control group, each of which was via a random priming method, and by using Klenow fragments (NimbleGen Dual-Color DNA Labeling Kit, Roche NimbleGen, Inc., Madison, WI, USA) according to the manufacturer’s protocol. Hybridization and scanning were performed according to the instructions in the NimbleGen Array User Guide. Data processing of the arrays was conducted by using Nimblegen’s standard protocol. Batch effects were removed with the ComBat R package. Quantile normalization was performed through the affy R package. During promoter methylation analyses of these three genes, normalized probeset values of the NimbleGen Human DNA Methylation 3x720K CpG Island Plus RefSeq Promoter Array (100718_HG18_CpG_Refseq_Prom_MeDIP) were used. For *SOX7*, normalized values of 39 probes located in chr8:10624714-10628519 (based on hg18) were summed for each of the diagnostic and relapse tumor samples for 20 MM cases to estimate promoter methylation levels. *PINX1* and *MSRA* promoter methylation analyses were performed similarly by summing normalized values of 29 probes (chr8:10734099-10737149) and 31 probes (chr8:9946799-9950043), respectively.

The RNA samples obtained from total DNA/RNA isolations for DNA copy number analysis were used for transcript expression analysis. Labeling and hybridizations were performed according to protocols from Affymetrix. Briefly, 100–300 ng of total RNA was amplified and labeled using the WT Sense Target labeling and control reagents kit (Affymetrix Inc., Santa Clara, CA, USA). Labeled RNA samples were then hybridized into the Human Gene 1.0 ST Array. After that, washing and scanning were performed using the GeneChip System (i.e. GeneChip Hybridization Oven 640, GeneChip Fluidics Station 450, and GeneChip Scanner 7G) (Affymetrix, Santa Clara, CA, USA). The expression value for each probe set was calculated using the Affymetrix Expression Console that uses the RMA (Robust Multi-Array Average) algorithm. Gene expression values generated with the Affymetrix Human Gene 1.0 ST arrays were utilized to identify mRNA expression values of these three genes. The array IDs for *SOX7*, *PINX1*, and *MSRA* mRNAs used for expression analyses are, respectively, as follows: 8149289, 8149296, and 8144557.

### 2.12. Evaluation of SOX7 mRNA Expression Levels Through Reanalyses of the Publicly Available GEP Data of Plasma Cell Neoplasms and Normal Plasma Cells

Gene expression profiling (GEP) data based on DNA microarray were assessed by using the NCBI GEO DataSet Browser (https://www.ncbi.nlm.nih.gov/gds, accessed on 16 April 2024). The experimental procedure and data processing are as follows: CD138-positive PCs were isolated using the AutoMACS separation system (Miltenyi-Biotec, Bergisch Gladbach, Germany) with the following purities: >95% for all MM and SMM cases, and >90% for MGUS patients as well as healthy donors. Total RNA was extracted from normal and tumor PCs using miRNeasy Mini Kit (Qiagen, Hilden, Germany) following the manufacturer’s protocol. The RNA integrity was assessed using an Agilent 2100 Bioanalyzer (Agilent, Palo Alto, CA, USA). Labeling and hybridizations were performed according to protocols from Affymetrix. Briefly, 100–300 ng of total RNA was amplified and labeled using the WT Sense Target labeling and control reagents kit (Affymetrix Inc., Santa Clara, CA, USA), followed by hybridization into the Human Gene 1.0 ST Array. Washing and scanning were then performed via the GeneChip System of Affymetrix (GeneChip Hybridization Oven 640, GeneChip Fluidics Station 450, and GeneChip Scanner 7G). During data processing, normalization was carried out by using the expression console (Affymetrix) with an RMA algorithm that involved background correction, normalization, and calculation of log2 converted expression values. As the myeloid contamination signature can be detected even in samples with high purity, those probes identifying genes exclusive of myeloid lineage were subtracted from the analysis at the beginning. The expression levels of *SOX7* for MM (*n* = 44) and high-risk SMM (*n* = 33) cases, as well as those of normal plasma cells of a previous publication [37], were obtained by searching this browser with the following dataset record: GDS4968. In this study (GSE47552), [HuGene-1_0-st] Affymetrix Human Gene 1.0 ST Array was used to measure the transcript expression profiles. During comparative analyses of gene expression data with the ‘Profile graph’ option of GEO2R (https://www.ncbi.nlm.nih.gov/geo/geo2r/, accessed on 16 April 2024), log2 transformed values were converted back to anti-log2 values. *SOX7* transcript expression was normalized to that of *RPL37A* or *EMC7* for each sample.

### 2.13. Evaluation of SOX7 mRNA Levels in Diagnostic and Relapsed MM Tumor Samples with qRT-PCR

Similar to the primers used for genomic qPCR, the qRT-PCR primer pairs were designed with the PrimerQuest software and obtained from Sentebiolab Biotech (Table S2B). After the testing of *SOX7* and *RPL37A* qRT-PCR primer pairs with gradient PCR, primer pairs that did not generate primer dimers and that gave specific bands were chosen. RNA samples isolated from diagnostic and relapsed MM patient samples were converted to cDNAs by using Quantitect Reverse Transcription Kit (Qiagen, Hilden, Germany). The specificity and efficiency of amplifications were evaluated with melting curve analyses and by running the amplicons in TAE agarose gels whenever needed. The following filtering criteria were applied during qRT-PCR analyses: If no amplification was observed with the *SOX7* primer pairs, and the Ct value of the housekeeping gene (i.e., *RPL37A*) amplifications was >30, these amplifications were not taken into account during calculations. If the Ct value of *RPL37A* amplifications was <30, *SOX7* Ct values were assumed as the latest cycle (i.e., 45) of the qPCR amplifications. If dimers were detected in any amplification, they were excluded from analyses. Relative levels of *SOX7* transcript expression in MM samples were normalized to those of the average of OSA-sorted PC samples.

### 2.14. Mutation Data of MM Samples

MM (Broad, Cancer Cell 2014) dataset related to a previously reported manuscript [19] was used in the cBioPortal website (https://www.cbioportal.org) to evaluate *SOX7* mutation(s) in MM cases. The type and frequency of *SOX7* mutations in MM cases (assessed on 3 April 2024) were visualized by generating a heatmap with the OncoPrint function. As an alternative mutation database, COSMIC browser (https://cancer.sanger.ac.uk/cosmic) was used to determine the frequency and type of *SOX7* mutations in MM. *SOX7* gene name was searched in COSMIC v99 (GRCh38). From the ‘Tissue distribution section’, ‘Hematopoietic and lymphoid’ option was chosen. After that, *SOX7* mutation information in MM was obtained by clicking on the ‘plasma cell myeloma’ link (assessed on 26 April 2024).

### 2.15. Evaluation of SOX7 mRNA Expression Levels in MM Cell Lines

RNA-Seq data of 1019 human cancer cell lines were assessed from the Cancer Cell Line Encyclopedia RNA-Seq data available in the Expression Atlas database (https://www.ebi.ac.uk/gxa/home). RNA-Seq expression data of *SOX7*, *CD38*, and *CD138* were visualized as FPKM for 29 MM cell lines available among these 1019 human cancer cell lines (assessed on 14 April 2024 and 22 April 2024). The raw data provider for this database is the NIH Genomic Data Commons.

### 2.16. Generation of Graphs and Statistical Analyses

The Excel application of Microsoft 365 (version 2403) (Microsoft Corp., Redmond, WA, USA) was used to generate bar graphics and box-whisker and scatter plots and perform statistical analyses. Pearson product moment correlation coefficient (r) values were calculated with the Pearson function. Statistical significances between different sample groups were evaluated by applying Student’s *t*-tests. *p* values less than 0.05 were considered statistically significant.

## 3. Results

### 3.1. SOX7 Deletions Are Frequent in Diagnostic and Relapsed MM Samples

**Figure 1:**
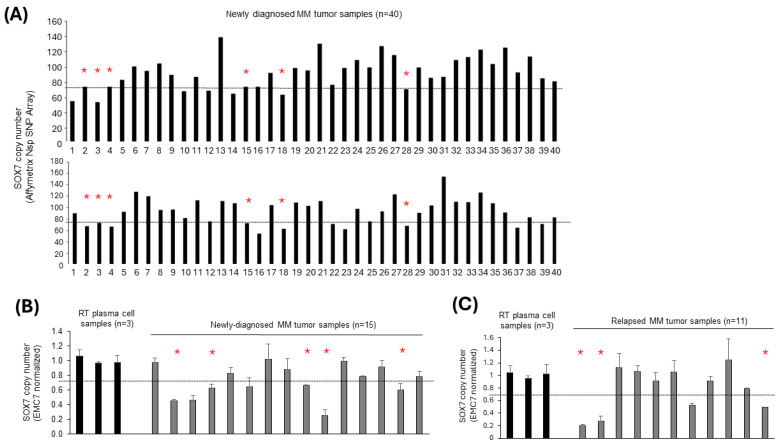
Recurrent *SOX7* deletions in newly diagnosed and relapsed MM tumors. (**A**) *SOX7* copy number in newly diagnosed MM cases based on the Affymetrix Mapping 250K Nsp SNP Array. The upper and lower panels show the results with the SNP_A-1859173 and SNP_A-1886065 probesets, respectively. The sample orders are the same for both panels. *SOX7* copy numbers were normalized to those of *RPL37A*, and each sample was normalized to patient-matched non-tumor DNA. MM cases having *SOX7* deletion based on both probesets are indicated with red stars in panel A (top and bottom). *SOX7* gene copy numbers in newly diagnosed (**B**) or relapsed (**C**) MM patient tumor samples based on qPCR. *EMC7* was used for normalization of *SOX7* gene copy numbers in qPCR reactions. Red stars in panels B and C indicate MM cases with *SOX7* deletion based on both *EMC7* and *RPL37A* (Figure S1) normalisations. Reactive tonsil PC samples were used as controls for normal copy number *SOX7*. RT: reactive tonsil.

To evaluate *SOX7* copy number status in MM cases, we analyzed *SOX7* copy number values based on SNP arrays in 40 newly diagnosed MM cases. Based on this analysis, *SOX7* deletion was present in at least 6 of 40 (15%) of the cases (Figure 1A). We also evaluated *SOX7* copy number status with qPCR of the in-house diagnostic and relapsed MM patient cohort. When each of two different reference genes located in different chromosomes (i.e., *EMC7* and *RPL37A*) were used for copy normalizations of the qPCR results, *SOX7* deletions were observed in 5 of 15 (33%) newly diagnosed MM samples compared to control PC samples (Figure 1B and Figure S1A). In tumor samples obtained at the relapse stage, 3 of 11 (27.3%) cases showed the presence of *SOX7* deletions in MM cases (Figure 1C and Figure S1B).

### 3.2. SOX7 Promoter Is Hypermethylated in Plasma Cell Malignancies

**Figure 2:**
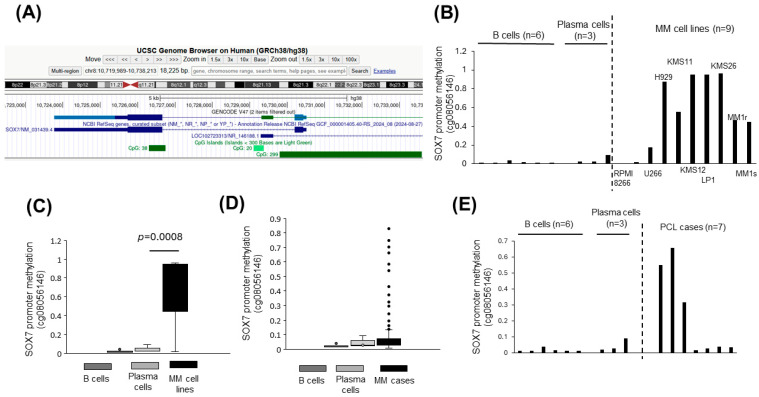
*SOX7* promoter is hypermethylated in MM. (**A**) The screen view showing the human *SOX7* gene locus with a blue color in the UCSC genome browser based on the current human reference genome. The direction of *SOX7* transcription is indicated in its intron from right to left according to NCBI RefSeq. Horizontal green bars indicate the CpG islands around the *SOX7* gene locus. Bar graphs (**B**) and the box-whisker (**C**) plot showing the *SOX7* promoter methylation status of normal B cell and PC samples as well as MM cell lines. (**D**) The box-whisker plot displaying the *SOX7* promoter methylation status in normal B cell samples (*n* = 6), PC samples (*n* = 3), and MM cases (*n* = 161). Outlier points are shown. (**E**) Bar graphs showing *SOX7* promoter methylation status in control (normal B cell and PC) samples and PCL cases. Dashed vertical lines in panels B and E separate cancer and normal samples. All the data are based on the Illumina HumanMethylation27 BeadChip probeset value for *SOX7* in the GSE21304 series. Plasma cells: CD138^+^ bone marrow cells; B cells: CD19^+^ peripheral blood cells. MM: multiple myeloma. PCL: plasma cell leukemia.

Based on the UCSC genome browser, there is a CpG island around the transcription start site of the human *SOX7* promoter (Figure 2A) raising the possibility of epigenetic downregulation of *SOX7* in MM. First, we analyzed the promoter methylation status of *SOX7* in MM cell lines and observed hypermethylation in 7 of 9 (77.8%) cell lines compared with that of normal PC or B cell samples (Figure 2B). Consistent with this observation, *SOX7* hypermethylation of MM cell lines was overall statistically significant (Figure 2C). At least, 13% (21 of 161) of the newly diagnosed MM cases showed hypermethylation of the *SOX7* promoter compared with that of the normal plasma or B cells (Figure 2D and Figure S2). Importantly, 3 of 7 (42.9%) of the PCL cases had hypermethylation of *SOX7* (Figure 2E). 

### 3.3. Deletion and Promoter Methylation of SOX7 Tend to Occur in Different MM Cases

**Figure 3:**
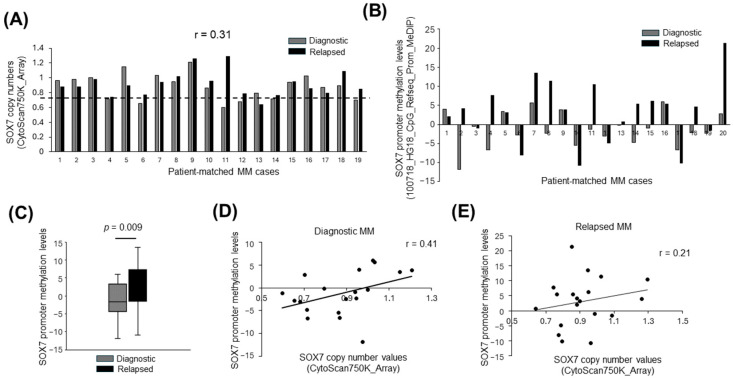
Deletion and promoter methylation as cooperative aberrations affecting different diagnostic or relapsed MM cases. Bar graphs showing the normalized gene copy number (**A**) or promoter hypermethylation (**B**) for *SOX7* in tumor samples obtained during diagnosis or relapse of patient-matched MM cases. The dashed horizontal line in panel A indicates the threshold for *SOX7* deletions. (**C**) The box-whisker plot of promoter methylation levels in diagnostic and relapsed MM cases. Scatter plots showing the correlation between *SOX7* copy numbers and promoter methylation levels in diagnostic (**D**) or relapsed (**E**) MM tumor samples. Pearson correlation r values indicate copy number correlations for patient-matched diagnostic and relapsed MM samples in panel A and correlations between *SOX7* copy numbers and promoter methylation values in panels (**D**,**E**).

Next, we evaluated *SOX7* deletion status in MM tumor samples obtained during the diagnosis or relapse stage. The copy number values of *SOX7* showed moderate levels of positive correlation between patient-matched diagnostic and relapsed MM samples (Figure 3A). *SOX7* promoter was methylated in 6 of 20 (30%) of the diagnostic MM cases, and 13 of 20 (65%) of the MM tumor samples obtained at relapse (Figure 3B). In fact, promoter methylation levels of *SOX7* were significantly higher in relapsed MM cases (Figure 3C). To address whether deletion and promoter hypermethylation of *SOX7* occur in the same or different cases, we analyzed the relationship between *SOX7* copy number and promoter methylation levels. Interestingly, *SOX7* copy number and promoter methylation values were positively correlated in diagnostic (Figure 3D) as well as relapsed (Figure 3E) MM cases.

### 3.4. The Influence of Deletion and Methylation on Expression of Candidate Tumor Suppressors in 8p23.1

**Figure 4:**
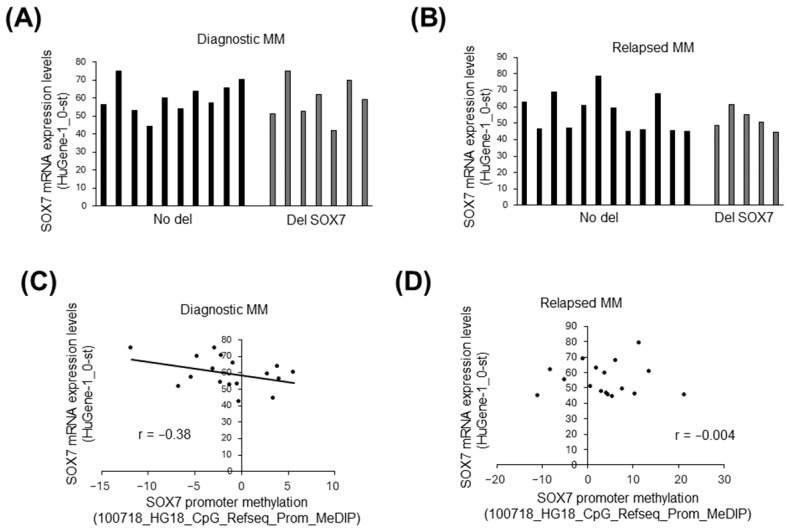
The relationship between deletion, methylation, and mRNA expression of *SOX7* in diagnostic and relapsed MM cases. Bar graphs showing *SOX7* mRNA expression levels in diagnostic (**A**) or relapsed (**B**) MM cases with or without *SOX7* deletion. Black bars represent cases with no deletion (No del), whereas gray bars represent MM cases with *SOX7* deletion based on CytoScan 750K arrays. Scatter plots comparing promoter methylation and mRNA expression levels of *SOX7* in patient-matched diagnostic (**C**) and relapsed (**D**) MM tumor samples. The trendline is shown in panel C. The array types relevant to each of these analyses are indicated within parentheses.

To address whether deletion or methylation affects mRNA expression levels of *SOX7* as well as other two tumor suppressor candidates located in del8p23.1, we first compared mRNA expression levels of *SOX7* with/without del8p23.1 in patient-matched diagnostic and relapsed MM samples. There was a marginal difference in terms of mRNA expression levels of diagnostic MM cases with/without *SOX7* deletion (Figure 4A and Figure S3A), whereas relapsed MM cases with *SOX7* deletion tended to have lower *SOX7* mRNA expression levels (Figure 4B and Figure S3B). We then compared promoter methylation and mRNA expression levels of *SOX7* in diagnostic and relapsed MM cases. There was an inverse correlation between promoter methylation and *SOX7* mRNA expression levels in diagnostic MM cases (Figure 4C); whereas, we did not observe any relationship in tumor samples obtained at relapse (Figure 4D).

**Figure 5:**
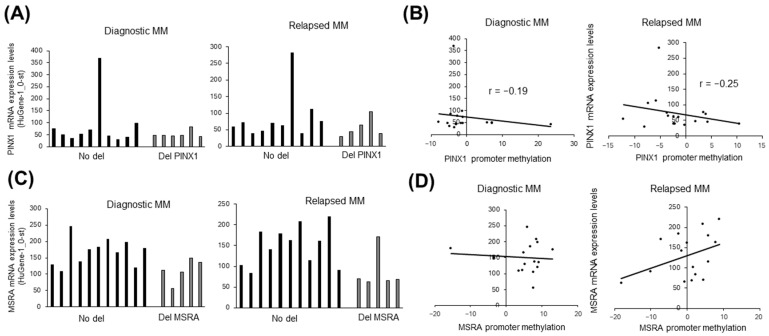
The relationship between deletion or promoter methylation with expression of *PINX1* and *MSRA* in MM cases. (**A**) Bar graphs showing *PINX1* mRNA expression levels of diagnostic (**left**) and relapsed (**right**) MM cases with/without *PINX1* deletion. (**B**) Scatter plots comparing promoter methylation and mRNA expression levels in diagnostic (**left**) and relapsed (**right**) MM cases. (**C**) Bar graphs showing *MSRA* mRNA expression levels of diagnostic (**left**) and relapsed (**right**) MM cases with/without *MSRA* deletion. (**D**) Scatter plots comparing promoter methylation and mRNA expression levels of *MSRA* in diagnostic (**left**) and relapsed (**right**) MM cases.

When we repeated the same analyses for *PINX1*, we observed that some MM cases without deletion have higher mRNA expression (Figure 5A). Of note, there was a moderate level of inverse correlation between promoter methylation and transcript expression (Figure 5B). The third candidate tumor suppressor gene (*MSRA*) evaluated showed lower mRNA expression in MM cases with deletion (Figure 5C), whereas there was no inverse correlation between methylation and expression (Figure 5D).

### 3.5. Mutations of SOX7 Are Infrequent in MM Tumor Samples

**Figure 6:**
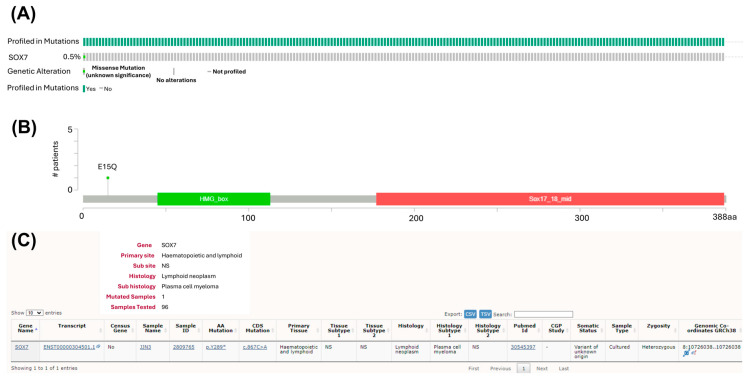
*SOX7* mutations are very rare in MM cases. (**A**) The oncoprint graph showing the only MM case with a *SOX7* mutation based on cBioPortal. (**B**) The lollipop plot showing the type and location of the identified *SOX7* mutation in the context of the primary protein structure. cBioPortal website was assessed on 3 April 2024 and 26 April 2024. The graphics are based on the mutation data in the Broad Cancer Cell 2014 manuscript. (**C**) A *SOX7* mutation identified in 1 of 96 plasma cell myeloma (multiple myeloma) samples based on the COSMIC database.

Next, we investigated whether small mutations (e.g., point mutations, indels) of *SOX7* were observed frequently in MM. To address this question, we first visualized the available *SOX7* mutation data in the cBioPortal database. Only one of the 205 MM samples (0.5%) evaluated contained a small mutation (Figure 6A), which was a missense mutation (p.E15Q) located close to the N-terminus in an MM case (Figure 6B). Next, we observed the *SOX7* mutation status of MM samples by browsing the COSMIC website. Similar to the available data in the cBioPortal database, the incidence of *SOX7* mutations in MM samples was quite low with a frequency of 1% (1 of 96 samples) (Figure 6C). The point mutation (c.867C>A) was located within the coding sequence of the JJN3 cell line with an expected alteration of p.Y289* in the SOX7 protein [38].

### 3.6. SOX7 Is Transcriptionally Silent in MM Cell Lines

**Figure 7:**
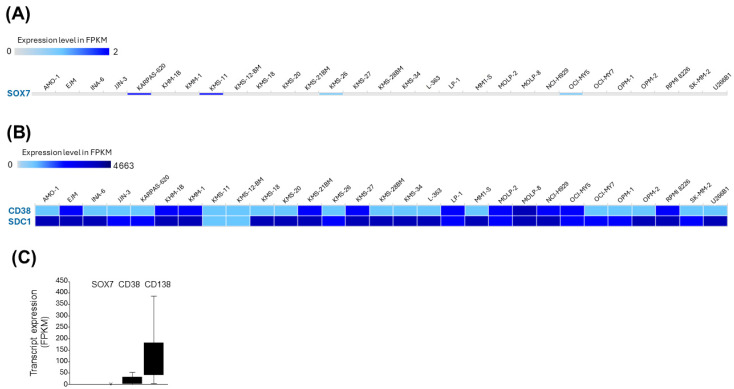
Lack of *SOX7* mRNA expression in MM cell lines. (**A**) Heatmap showing *SOX7* transcript levels in MM cell lines. (**B**) Heatmap showing CD38 and SDC1 (CD138) transcript levels in MM cell lines. (**C**) The box-whisker graphic showing *SOX7* transcript levels in comparison to CD38 and CD138. FPKM: fragments per kilobase of transcript per million mapped reads.

When RNA-seq expression levels of 29 MM cell lines from the Cancer Cell Line Encyclopedia were visually analyzed in the Expression Atlas database, we observed that 25 of 29 (86.2%) MM cell lines did not have *SOX7* transcript expression, whereas other MM cell lines also had quite low expression (Figure 7A). By contrast, two specific markers of MM, *CD38* and *CD138* (*SDC1*), were expressed in all MM cell lines (Figure 7B). Compared with the expression levels of *CD38* and *CD138* (*SDC1*), *SOX7* can be considered transcriptionally silent in all MM cell lines (Figure 7C).

### 3.7. SOX7 Was Underexpressed in MM and High-Risk SMM Compared to Normal PCs

**Figure 8:**
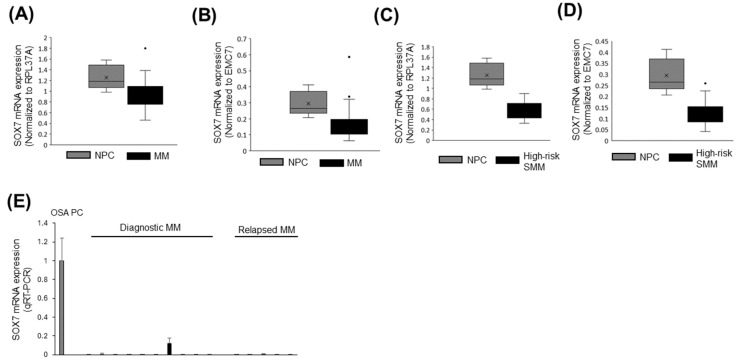
SOX7 is downregulated in MM and high-risk SMM cases. Box-whisker plots comparing *SOX7* mRNA expression in normal PC samples and MM case tumor samples normalized to that of RPL37A (**A**) or EMC7 (**B**) as the housekeeping gene. Box-whisker plots comparing *SOX7* mRNA expression in normal PC samples and high-risk SMM case tumor samples normalized to that of RPL37A (**C**) or EMC7 (**D**) as the housekeeping gene. Means and outlier samples are indicated with crossmarks and dots, respectively, in box-whisker plots. NPC: normal plasma cell. MM: multiple myeloma. SMM: smoldering multiple myeloma. (**E**) The bar plot showing relative *SOX7* mRNA expression in OSA plasma cells as well as diagnostic and relapsed MM cases based on qRT-PCR analysis. RPL37A was used as the housekeeping gene for the normalization of *SOX7* mRNA expression. Means ± std of replicates are shown. OSA: obstructive sleep apnea. PC: plasma cell.

Next, we analyzed previously reported DNA microarray data to evaluate whether *SOX7* is differentially expressed in MM cases compared to normal PCs. We observed significant downregulation of *SOX7* expression in MM bone marrow tumor samples compared to normal bone marrow PC samples when expression levels of RPL37A (Figure 8A) or EMC7 (Figure 8B) housekeeping gene were used to obtain normalized expression of each sample. Using the same DNA microarray data, we also analyzed *SOX7* transcript expression in high-risk SMM cases. Similar to the results of MM cases, *SOX7* transcript levels were significantly lower in high-risk SMM cases compared with control samples regardless of whether RPL37A (Figure 8C) or EMC7 (Figure 8D) was used for normalization of the *SOX7* gene expression across the samples. Similar to the results of the DNA microarray, we observed underexpression of *SOX7* by qRT-PCR in diagnostic and relapsed MM cases compared with the control group samples (Figure 8E).

## 4. Discussion

Genomic regions with recurrent deletions are enriched in tumor suppressor genes [39]. Identification of these deleted regions by conducting genome-wide copy number analyses [40] generates a short list of candidate tumor suppressors for further studies and functional characterization [41,42]. Similar to other high-throughput genome-wide approaches, the copy number alterations identified by CGH or SNP arrays [43] need to be cross-validated with locus-specific approaches such as qPCR or FISH [44] before proceeding with further characterization.

Both SNP array and quantitative PCR results provided support for the presence of *SOX7* deletions in a fraction of MM cases (Figure 1), which is in line with a previous study reporting homozygous deletions of 8p23.1 genomic locus in certain MM cases [19]. The fact that *SOX7* deletions are also frequent in relapsed MM cases supports a previous study that reported *SOX7* to be deleted in an MM case during two consecutive relapses [29]. Given that lower expression levels were observed in many relapsed MM cases with *SOX7* deletion both in the public data (Figure 4B) and the in-house cohort (Figure S3B), *SOX7* underexpression due to this deletion may be one of the contributing factors for clonal selection associated with MM relapse. Tumor heterogeneity due to the presence of multiple genetic clones [45] together with the presence of variable degrees of promoter hypermethylation among MM tumor samples may be responsible for the less obvious difference in *SOX7* transcript expression in MM cases with/without delSOX7.

Epigenetic silencing through promoter-associated CpG island hypermethylation is a commonly observed alteration leading to silencing of tumor suppressor genes in a variety of different lymphoid cancer types such as diffuse large B cell lymphoma [46], natural killer cell lymphoma [47], and mantle cell lymphoma [48]. In general, hypermethylation-mediated silencing occurs through the recruitment of histone deacetylases [49] or nucleosome remodeling complexes [50], which, in turn, generate transcriptionally repressive chromatin.

Since *SOX7* promoter hypermethylation is frequently observed in MM cell lines (Figure 2B,C) and MM cases (Figure 2D), *SOX7* inactivation in MM may be mainly through the cooperation of deletion and promoter hypermethylation instead of small mutations that are infrequent. The observation that promoter-associated *SOX7* hypermethylation is also frequent in PCL cases (Figure 2E) suggests *SOX7* as a mainly epigenetically silenced tumor suppressor not only in MM but also in PCL. Future research focusing on the role of *SOX7* promoter methylation-related transcriptional cofactors in the *SOX7* locus may reveal epigenetic regulators as therapy targets for plasma cell malignancies. Given the positive correlation between copy numbers and methylation (Figure 3D), promoter hypermethylation may be responsible for *SOX7* underexpression in newly diagnosed MM cases without deletion. However, we cannot totally exclude the contribution of other mechanisms leading to the underexpression of *SOX7* observed in most of the MM cases (Figure 8).

Although these observations provide further evidence that *SOX7* is, probably, the most likely candidate gene deserving further investigation in del8p23.1 as a tumor suppressor, we cannot exclude the possibility that *PINX1* or *MSRA* may also have tumor suppressor role owing to their downregulation through deletion and/or promoter methylation in MM cases (Figure 5). Future studies will elucidate whether these genes play a role in MM tumorigenesis or relapse.

There are certain limitations of this study that can be summarized as follows: Firstly, the number of MM cases evaluated for *SOX7* copy number with qPCR is relatively small. Secondly, the promoter hypermethylation results are based on microarray data, which may need validation with a locus-specific approach such as pyrosequencing [41] for more refined values. Thirdly, the number of patient-matched samples of MM cases used for integrative analyses of copy number, methylation, and transcription is relatively low. Given the heterogeneity of tumor samples, analyses of *SOX7* copy number, promoter methylation, and transcript levels integratively at the single cell level with NGS-based methodologies [51] may yield more refined results regarding the relationship between these genetic or epigenetic aberrations and *SOX7* mRNA expression. Finally, other possible mechanisms, such as miRNA-mediated silencing leading to lower expression of SOX7, were not investigated in this study. Future studies may include comprehensive functional characterization of *SOX7* in MM cell lines with ectopic *SOX7* expression through cell cycle and apoptosis assays. It will also be interesting to identify transcriptional targets of *SOX7* in MM cell lines through methodologies such as ChIP-Seq and RNA-Seq to address whether *SOX7* targets genes related to tumor suppression.

## 5. Conclusions

In conclusion, genetic and/or epigenetic inactivation of *SOX7* may play a role during the development of MM and allied diseases such as SMM or PCL. Future functional studies will shed light on not only the role of *SOX7* in MM cancerogenesis but also on its role as a potential diagnostic or prognostic biomarker. If sufficient evidence for *SOX7* can be obtained supporting a tumor suppressive role through functional studies, re-expression of silenced *SOX7* through the administration of epi-drugs such as HDAC inhibitors [52] can be considered as a targeted therapy option for MM and allied neoplasms.

## Data Availability

The accession numbers of publicly available data used in this study are available in the Section 2. The raw data of the in-house experiments can be requested as long as there is a reasonable justification.

## Supplementary Materials

The following supporting information can be downloaded at: https://www.mdpi.com/article/10.3390/cimb47040244/s1.

## Abbreviations

The following abbreviations are used in this manuscript:

MM	Multiple myeloma
SMM	Smoldering multiple myeloma
PCL	Plasma cell leukemia
CRC	Colorectal cancer
QC	Quality check
ACK	Ammonium–chloride–potassium
MACS	Magnetic-activated cell sorting
GEP	Gene expression profiling
OSA	Obstructive sleep apnea
HDAC	Histone deacetylase
NGS	Next-generation sequencing
