# Supplementary material for: Genetic and Epigenetic Aberrations of SOX7 in Newly Diagnosed and Relapsed Multiple Myeloma as Well as Related Neoplasms
Source: Curr Issues Mol Biol. 2025 Apr 1;47(4):244. doi: 10.3390/cimb47040244 (PMC12026369; doi:10.3390/cimb47040244)
Supplement: Supplementary file 1 [file cimb-47-00244-s001.zip › Supplementary Figures (SOX7 MM Diagnostics) 26.02.25 .pdf]

Figure S1

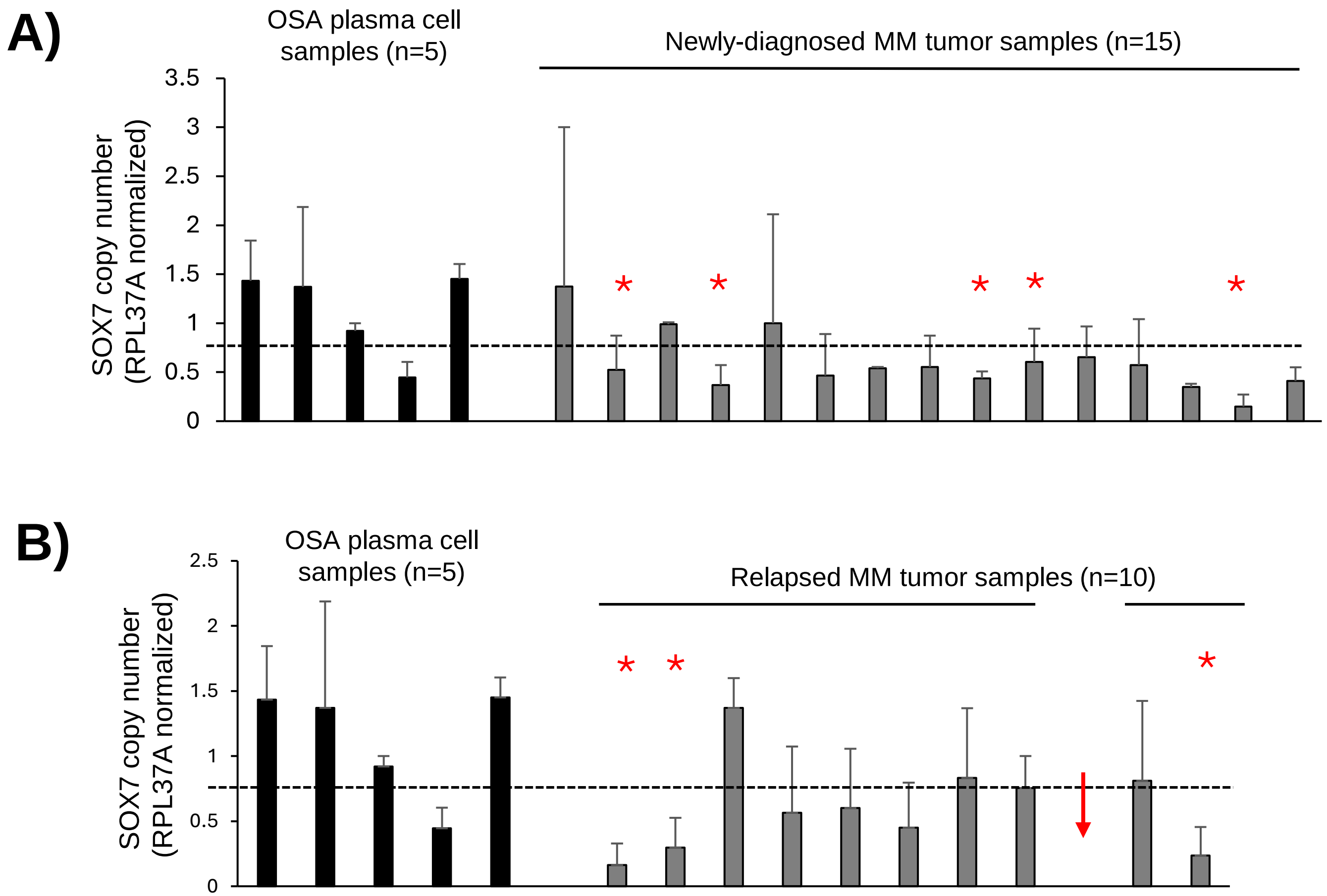

SOX7 copy number evaluation with qPCR based on normalization to RPL37A reference gene. A) *SOX7* gene copy number in newly diagnosed MM patient tumor samples; **B**) SOX7 gene copy numbers in relapsed multiple myeloma tumor samples. RPL37A gene was used for normalization of SOX7 gene copy numbers in qPCR reactions. Obstructive sleep apnea tonsil plasma cell samples were used as the controls for normal copy number SOX7. Horizontal dashed lines are the thresholds for considering an MM sample to have deletion. Red stars indicate the MM cases with SOX7 deletion. The order of the indicated MM cases are the same as Figure 1 for both diagnostic and relapsed MM cases. Red arrow indicates the 9th relapsed MM case with no available qPCR data.

Figure S2

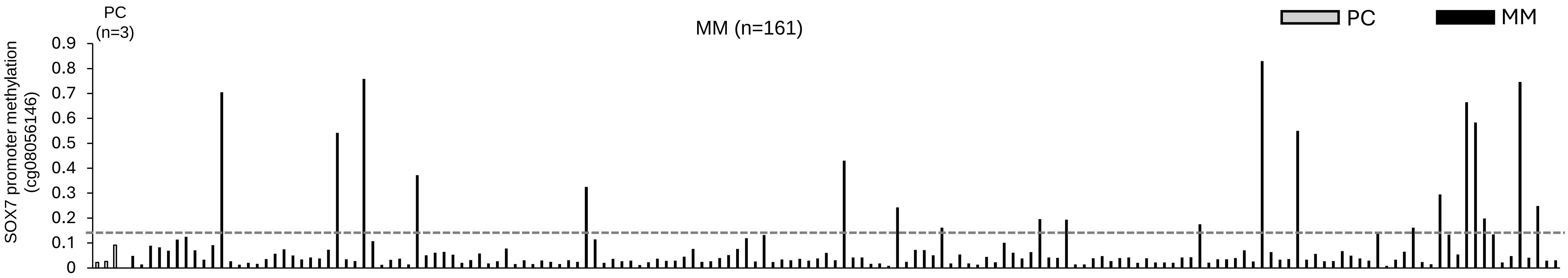

Comparison of promoter methylation levels of SOX7 in MM cases and normal plasma cells. Promoter methylation levels of SOX7 are shown for tumor samples of 161 MM cases and three non-cancer plasma cell samples as controls. Horizontal dashed lines show the threshold used to determine whether a MM tumor sample methylated or not. The probe sets used to evaluate methylation are shown in the y axis for SOX7. PC: Normal bone marrow plasma cell.

Figure S3

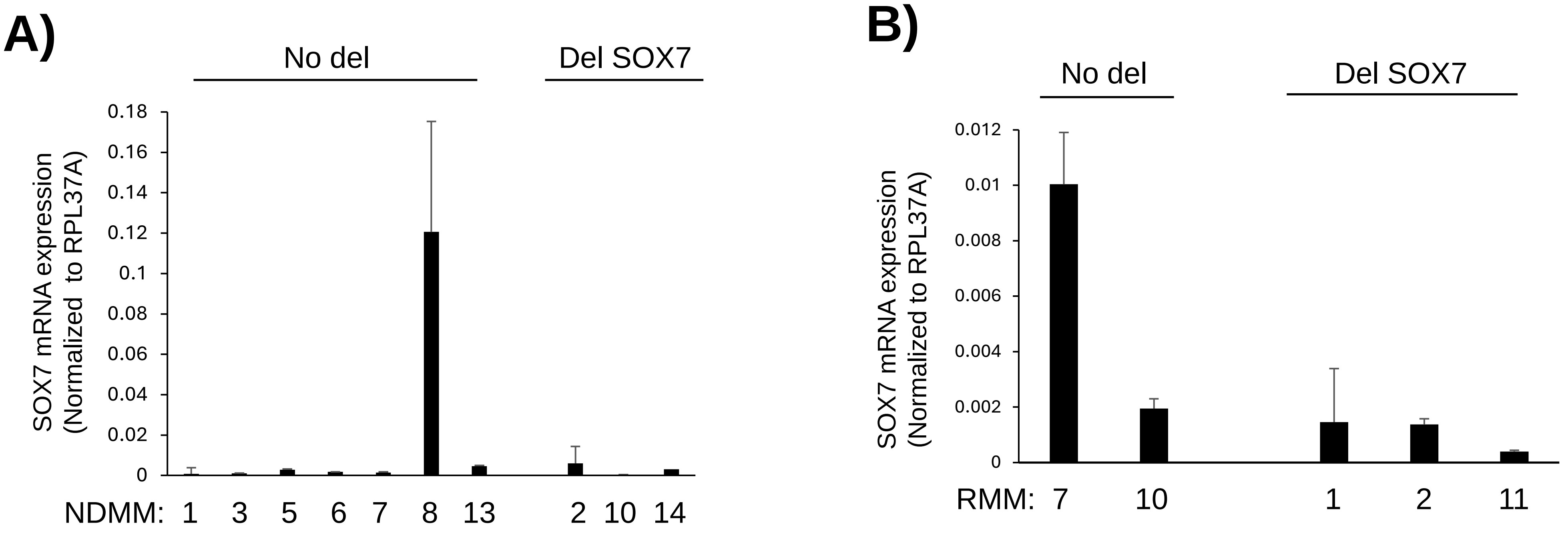

Comparison of SOX7 mRNA expression with qRT-PCR in MM cases with or without SOX7 deletion. **A)** SOX7 mRNA expression in diagnostic MM cases with/without SOX7 deletion. **B)** SOX7 mRNA expression in relapsed MM cases with/without SOX7 deletion. NDMM: Newly diagnosed multiple myeloma; RMM: Relapsed multiple myeloma. The relative values of SOX7 in each MM case is calibrated to that of OSA plasma cell sample. Numbers below plots indicate patient number. Means ± SD of replicate measurements are shown.
