# Supplementary material for: Genetic and Epigenetic Aberrations of SOX7 in Newly Diagnosed and Relapsed Multiple Myeloma as Well as Related Neoplasms
Source: Curr Issues Mol Biol. 2025 Apr 1;47(4):244. doi: 10.3390/cimb47040244 (PMC12026369; doi:10.3390/cimb47040244)
Supplement: Supplementary file 1 [file cimb-47-00244-s001.zip › Table S1. Diagnosed and relapsed MM patient information (Diagnostics) 22.02.25 .pdf]

Table S1. Demographic and clinicopathological characteristics of diagnosed and relapsed MM cases.

	Diagnosed MM cases (N=15)	Relapsed MM cases (N=13)
**Gender**		
Male	10 (66.6%)	9 (69.2%)
Female	5 (33.3%)	4 (30.8%)
**Age (years)**		
Mean (SD)	64.6 (6.2)	62.5 (11.3)
Median [Min, Max]	63 [52, 78]	63 [39, 80]
**Quantitative Serum Immunoglobulins**		
**IgG**		
Mean (SD)	2,629.82 (2,426.10)	2,417.13 (1,945.29)
Median [Min, Max]	902.52 [298.75, 6,676.76]	2,153 [206.4, 6,558]
**IgA**		
Mean (SD)	570.91 (1,324.86)	470.73 (638.10)
Median [Min, Max]	65.90 [7.81, 4,852.20]	215 [9, 2,178]
**IgM**		
Mean (SD)	36.01 (18.58)	40.74 (32.74)
Median [Min, Max]	32.17 [15.26, 80.34]	30.50 [4.96, 108]
**Hemoglobin Level (gr/dL)**		
Mean (SD)	10.87 (1.53)	11.00 (2.18)
Median [Min, Max]	10.60 [8.60, 14.30]	10.80 [6.40, 15.10]
**Creatinine Level (mg/dL)**		
Mean (SD)	2.01 (2.04)	1.22 (0.67)
Median [Min, Max]	1.16 [0.57, 8.49]	0.87 [0.69, 3.32]
**GFR (ml/min)**		
Mean (SD)	57.67 (31.30)	66.54 (23.18)
Median [Min, Max]	64 [4, 108]	69 [20, 106]
**Calcium Level (mg/dL)**		
Mean (SD)	10.31 (1.02)	9.25 (0.57)
Median [Min, Max]	10.00 [8.61, 12.40]	9.30 [8.16, 9.90]
**Albumin Level (g/dL)**		
Mean (SD)	3.58 (0.73)	3.82 (0.57)
Median [Min, Max]	3.92 [2.50, 4.70]	3.90 [2.93, 4.88]
**LDH Level (U/dL)**		
Mean (SD)	164.67 (46.08)	158.31 (42.60)
Median [Min, Max]	155 [84, 241]	144 [99, 259]
**β2 Microglobulin Level (ng/mL)**		
Mean (SD)	12.25 (19.33)	6.02 (2.72)
Median [Min, Max]	4.20 [0.53, 67.90]	6.09 [1.98, 10.64]
**Bone Marrow Plasma Cell Percentage (%)**		
Mean (SD)	54.00 (20.35)	52.55 (22.47)
Median [Min, Max]	50 [10, 100]	50 [18, 90]

GFR: Glomerular Filtration Rate

LDH: Lactate Dehydrogenase
