# Supplementary material for: Genetic and Epigenetic Aberrations of SOX7 in Newly Diagnosed and Relapsed Multiple Myeloma as Well as Related Neoplasms
Source: Curr Issues Mol Biol. 2025 Apr 1;47(4):244. doi: 10.3390/cimb47040244 (PMC12026369; doi:10.3390/cimb47040244)
Supplement: Supplementary file 1 [file cimb-47-00244-s001.zip › Table S2. qPCR and qRT-PCR primers (Diagnostics) 22.02.25 .pdf]

Table S2. **Primers used for genomic qPCR and qRT-PCR experiments.**

A) The list of genomic qPCR primers used to evaluate SOX7 copy number

	**Forward primer**	**Reverse primer**
**SOX7**	5’-GTTAGGACACACCCGAACAA-3’	5’-CCTCGTCGAAAGGCAAAGA-3’
**EMC7**	5’-CCTGAAGGAAGAGGAGAGTAGA-3’	5’-CCTCTGACTGGCTGTGTTAAG-3’
**RPL37A**	5’-GCTTTCACATCCCTGACAATAAG-3’	5’-CTCGTCTCTTCATCTTGGTCTATAA-3’

B) The list of qRT-PCR primers used to evaluate SOX7 mRNA expression

	**Forward primer**	**Reverse primer**
**SOX7-1st**	5’-TAGAGCAACTTCCCGCAAATC-3’	5’-ACACTGTTATGCATGCTCCTTTA-3’
**SOX7-2nd**	5’-GCCGAGCTCAGCAAGAT-3’	5’-CGGCCGGTACTTGTAGTTG-3’
**RPL37A**	5’-AGTCGGGATCGTCGGTAAATA-3’	5’-GCCACAGAAAGAGCAAGTGTA-3’
